# Maternal diabetes and obesity influence the fetal epigenome in a largely Hispanic population

**DOI:** 10.1186/s13148-020-0824-9

**Published:** 2020-02-19

**Authors:** Heather E. Rizzo, Elia N. Escaname, Nicholas B. Alana, Elizabeth Lavender, Jonathan Gelfond, Roman Fernandez, Matthew A. Hibbs, Jonathan M. King, Nicholas R. Carr, Cynthia L. Blanco

**Affiliations:** 1 Department of Biology, Trinity University, 1 Trinity Place, San Antonio, TX 78212 USA; 2 Pediatrics, University of Texas Health Science Center San Antonio, San Antonio, TX USA; 3 University Health System, San Antonio, TX USA; 4 Department of Neonatal Medicine, Brooke Army Medical Center, San Antonio, TX USA

**Keywords:** Epigenetics, DNA methylation, Obesity, Diabetes mellitus, Metabolic programming, Newborn

## Abstract

**Background:**

Obesity and diabetes mellitus are directly implicated in many adverse health consequences in adults as well as in the offspring of obese and diabetic mothers. Hispanic Americans are particularly at risk for obesity, diabetes, and end-stage renal disease. Maternal obesity and/or diabetes through prenatal programming may alter the fetal epigenome increasing the risk of metabolic disease in their offspring. The aims of this study were to determine if maternal obesity or diabetes mellitus during pregnancy results in a change in infant methylation of CpG islands adjacent to targeted genes specific for obesity or diabetes disease pathways in a largely Hispanic population.

**Methods:**

Methylation levels in the cord blood of 69 newborns were determined using the Illumina Infinium MethylationEPIC BeadChip. Over 850,000 different probe sites were analyzed to determine whether maternal obesity and/or diabetes mellitus directly attributed to differential methylation; epigenome-wide and regional analyses were performed for significant CpG sites.

**Results:**

Following quality control, agranular leukocyte samples from 69 newborns (23 normal term (NT), 14 diabetes (DM), 23 obese (OB), 9 DM/OB) were analyzed for over 850,000 different probe sites. Contrasts between the NT, DM, OB, and DM/OB were considered. After correction for multiple testing, 15 CpGs showed differential methylation from the NT, associated with 10 differentially methylated genes between the diabetic and non-diabetic subgroups, CCDC110, KALRN, PAG1, GNRH1, SLC2A9, CSRP2BP, HIVEP1, RALGDS, DHX37, and SCNN1D. The effects of diabetes were partly mediated by the altered methylation of HOOK2, LCE3C, and TMEM63B. The effects of obesity were partly mediated by the differential methylation of LTF and DUSP22.

**Conclusions:**

The presented data highlights the associated altered methylation patterns potentially mediated by maternal diabetes and/or obesity. Larger studies are warranted to investigate the role of both the identified differentially methylated loci and the effects on newborn body composition and future health risk factors for metabolic disease. Additional future consideration should be targeted to the role of Hispanic inheritance. Potential future targeting of transgenerational propagation and developmental programming may reduce population obesity and diabetes risk.

## Background

Childhood obesity and diabetes mellitus are an increasing epidemic in the USA [1]. In 2015, an estimated 30.3 million people in the USA had diabetes mellitus (DM). Approximately 12.7 million children and adolescents ages 2 to 19 are obese, and it is estimated that > 25% of children will be classified as overweight or obese by kindergarten [2]. Hispanic Americans are particularly at risk for obesity, diabetes, and end-stage renal disease [3]. The risk of obesity is 35% higher in the Hispanic population, with obese Hispanic and non-Hispanic black adolescent females among those at highest risk of developing type II diabetes [1, 4]. Concomitant obesity and diabetes during pregnancy are also associated with increased risk of metabolic syndrome in the offspring [5]. In South Texas alone, 29% of mothers have a pre-pregnancy BMI of 30 or above, and 4.8% of mothers go on to develop gestational diabetes (GDM) [6]. This study seeks to investigate whether previously identified and unidentified associations occur between maternal diabetes, obesity, and altered newborn methylation in an already high-risk Hispanic population of South Texas.

The combination of obesity and gestational diabetes mellitus is estimated to complicate up to 9.2% of pregnancies, with the highest risks for gestational diabetes affecting ethnic and racial minority women [7]. Exposure to a diabetic intrauterine environment during pregnancy is associated with an increase in dyslipidemia, subclinical vascular inflammation, and endothelial dysfunction processes in the offspring, all of which are linked with development of cardiovascular disease later in life [8]. Maternal obesity and gestational diabetes have additionally been linked to increased risk of asthma, poorer cognitive performance, mental health disorders, neurodevelopmental disorders including cerebral palsy, and immune and infectious disease-related outcomes [9].

Increasing evidence has shown that transgenerational non-genetic inheritance can occur through in utero exposure of the developing fetus to the maternal environment or through either the male or female germline [10]. The concept of “gestational programming” is associated with alterations to the epigenome (non-genomic) as opposed to alteration in the genomic DNA sequence [11–13]. Significant hypermethylation of DNA may also occur globally in the placenta of mothers with GDM as well as the cord and neonatal blood of infants born to mothers with GDM, particularly genes associated with metabolic disease [14–17]. This hypermethylation may repress transcription leading to dysregulation of metabolic pathways. Epigenetic mechanisms may contribute to altered beta cell mass and beta cell failure, similarly as observed in diabetes [18]. Pregnancy complications with fetal exposure to glucocorticoids, either from maternal stress or synthetic glucocorticoids, can also lead to prolonged alteration of hypothalamic-pituitary-adrenal function [19, 20].

While pre-pregnancy maternal obesity is associated with adverse offspring outcomes at birth and later in life, the role of pre-pregnancy BMI is less clear [21]. The Pregnancy and Childhood Epigenetics (PACE) Consortium found a causal intrauterine effect of maternal BMI on newborn methylation at just 8/86 sites in a recent meta-analysis, attributing the identified robust associations between maternal adiposity and variations in DNA methylation to genetic or lifestyle factors [22]. Additionally, abnormal maternal nutrition, diet, folic acid, and vitamin deficiency can induce epigenetic alterations including DNA methylation, histone modifications, chromatin remodeling, and/or regulatory feedback by microRNAs, all of which have the ability to modulate gene expression and promote a metabolic syndrome phenotype [23–28].

We conducted epigenetic analysis via epigenome-wide association studies (EWAS) and regional analysis targeting genes associated with infant insulin signaling, glucose metabolism, and free fatty acid pathways in term infants delivered to mothers with normal weight, obesity, and DM in a highly Hispanic population. There are clear disparities in the risk of gestational diabetes by race and ethnicity, and small population studies in high-risk ethnicities are lacking. Our primary analyses focused on identifying areas of significant methylation differences between diabetic and non-diabetic Hispanic populations and obese and non-obese Hispanic populations. Additional analyses were also performed to determine whether the observed mediation effects were related to diabetes alone, obesity alone, or in combination.

## Methods

### Sample description

The study protocol was approved by the Institutional Review Board at The University of Texas Health Science Center, San Antonio (IRB# HSC 2016 0097H). Infants were prospectively enrolled between the years of 2016–2018. Limited neonatal data were available for extrapolation and determination of an ideal sample size. Complementary enrollment in a body composition analysis with the same inclusion/exclusion criteria occurred during study completion. A priori power analysis was completed for body composition analysis enrolling competitive patient enrollment in this cohort. Initial intention was prospective enrollment of 10 patients from each targeted population [29] but was expanded to 23 per arm (69) given to variability within targeted populations as well as to maximize efficiency of array utilization.

### Population

All babies ≥ 37 weeks gestational age and ≤ 48 h old who were admitted to the newborn nursery at the University Hospital in San Antonio, TX, and who were eligible for inclusion were screened for recruitment. Inclusion criteria for enrollment included infants with and without a maternal history of diabetes mellitus (DM) and obesity (OB). Maternal diabetes mellitus was defined as abnormal glucose tolerance test during current pregnancy +/− medical therapy to include insulin or other antidiabetic agents or diagnosis of type I or type II diabetes prior to current pregnancy. Maternal obesity was defined as BMI ≥ 30. Exclusion criteria included infants with congenital anomalies, complex congenital heart disease, and severe central nervous system disease (grade 4 intraventricular hemorrhage; malformations). Infants requiring supplemental oxygen or admission to the neonatal intensive care unit for any reason were also excluded. Infants of mothers with DM and/or OB were identified through the electronic medical record. Eligible participants were actively recruited after screening for inclusion/exclusion criteria and confirmed willingness to participate. Written informed consent was obtained from all participating mothers prior to enrollment. Potential subjects were contacted within 24 h of delivery with the cord blood obtained upon delivery.

### Blood collection, DNA extraction, and genome-wide methylation assay

Whole cord blood samples of 3–5 mL were collected immediately after birth. Blood samples were processed via centrifugation with 4 mL CPT BD Vacutainer tubes within 24 h to isolate and enriched for agranular leukocytes. DNA extraction was performed using the DNeasy Blood and Tissue Kit DNA kit (Qiagen). Isolated DNA was treated with a bisulfite conversion and run on Illumina MethylationEPIC BeadChip 850K array (UT Health SA Genomics Core).

### Statistical analysis

We calculated means and standard deviations (SD) for all maternal and newborn characteristics to describe the study population overall. We additionally examined maternal pre-pregnancy BMI, age at enrollment, gestational diabetes status, and mode of delivery as potential confounders. Maternal covariates were added to all final models if they were associated with any of the log-transformed outcomes in linear regression models at *p* < 0.05.

### Applied software

Quality control (QC) and all statistical analyses were performed using the R version 3.5.2 statistical analysis software, and the R-packages SWAN, missmethyl, minfi, limma, IlluminaHumanMethylation450kanno.ilmn12.hg19, IlluminaHumanMethylation450kmanifest, IlluminaHumanMethylationEPICmanifest, IlluminaHumanMethylationEPICanno.ilm10b2.hg19, bumphunter, RColorBrewer, matrixStats, minfiData, Gviz, DMRcate, and stringr.

### Data preprocessing, QC, and filtering

Detection *p* values were calculated for all samples with removal of samples below 0.05 from the data set. getQC and plotQC were used to estimate quality of samples as well. Subset-quantile within array normalization (SWAN) was used to normalize the data taking into account the fact that the array (EPIC methylation bead chip) contained two different types of probes. Additional quality controls were completed after normalization: (1) Detection *p* values were again used to remove any probes which failed in one or more samples (7169 probes). (2) Additional probes on sex chromosomes were removed to reduce sex-linked variation in methylation between the samples (18,975 probes). (3) Probes associated with single-nucleotide polymorphisms (SnP) were removed using dropLociWithSnPs from the minfi package (28,179 probes). (4) Probes known to be cross-reactive within the methylation probe set were removed (38,756 probes). After technical QC, a total of 773,012 sites and 69 individuals were in principle available for analysis. All images and data were validated by GenomeStudio as a quality control measure.

### Data transformation

After normalization, the data were processed to calculate beta values (methylated probe intensity at that site, over the total probe intensity of both the methylated and unmethylated probes) and *M* values (log2 of the methylated probe intensity over the unmethylated probe intensity). When performing statistical tests, *M* values were utilized as beta values tend to have heteroscedasticity; beta values were used for visualization in the figures and plots [30].

### Epigenome-wide association study

To find differentially methylated positions or probes, several methods were employed. Differentially methylated probes were identified using lmfit and ebayes in R [29]. Descriptors of either diabetic or non-diabetic, or obese or non-obese, were used under lmfit to design a linear fit to model the data. ebayes was then used to determine significant methylation values between the groups. Statistical significance for genome-wide associations was adjusted for multiple comparisons using a false discovery rate (Benjamini-Hochberg correction method) *q* < 0.05.

### Regional analyses

We examined the association of diabetes and/or obesity with differentially methylated regions (DMRs) in the cord blood using the R Bioconductor package bumphunter. Instead of employing a probe-wise approach, clusters of probes in the array are identified using the function clusterMaker, and the bumphunter function is used to fit a linear model accounting for the given sample variables to each identified cluster of probes in the given length (1000 bp) to determine if a region has a significant change in methylation compared with the control group. For our analysis, 1000 permutations were performed [31].

## Results

Descriptive statistics of the study population are shown overall and stratified by maternal comorbidities in Table 1. Mothers had a mean age of 29.3 (SD = 5.6) at enrollment and a mean BMI of 29.5 (SD = 4.2). An overall Hispanic predominance in patient ethnicity was seen across all groups. There were significant increases in age and BMI for both the diabetic (DM) and obese-diabetic (OB/DM) groups compared with the non-diabetic healthy weight (NT). There was an increased rate of cesarean section delivery for the obese (OB) and OB/DM mothers. Birth weight, length, and occipital-frontal circumference were similar between all groups.

**Table 1:** Maternal and infant demographic metrics

	Non-diabetic healthy weight	Diabetic healthy weight	Non-diabetic obese	Diabetic obese
Maternal age^a^	27.4 (6.3)	32.1 (5.5)	27.6 (5.0)	33.8 (5.4)
Maternal pre-pregnancy BMI^a^	23.82 (3.26)	26.24 (1.93)	35.31 (6.21)	34.58 (4.75)
Ethnicity (*n*)				
Hispanic	16	10	22	9
Non-Hispanic white	3	1	0	0
African American	2	1	1	0
Other	2	2	0	0
Infant born to condition (*n*)	23	14	23	9
C-section (%)	17%	29%	30%	44%
Birth weight in grams^a^	3464 (463)	3405 (348)	3764 (465)	3809 (474)
Gestational age at birth^a^	39.8 (1.1)	38.7 (0.8)	40.0 (1.0)	39.3 (1.5)
Birth length in centimeter^a^	51.3 (2.4)	50.9 (1.8)	52.1 (2.4)	50.9 (1.8)
Frontal-occipital circumference in centimeter^a^	34.5 (1.1)	34.4 (0.9)	35.1 (1.1)	35.2 (1.2)

^a^Mean values (standard deviation)

### Epigenome-wide association study

**Fig. 1:**
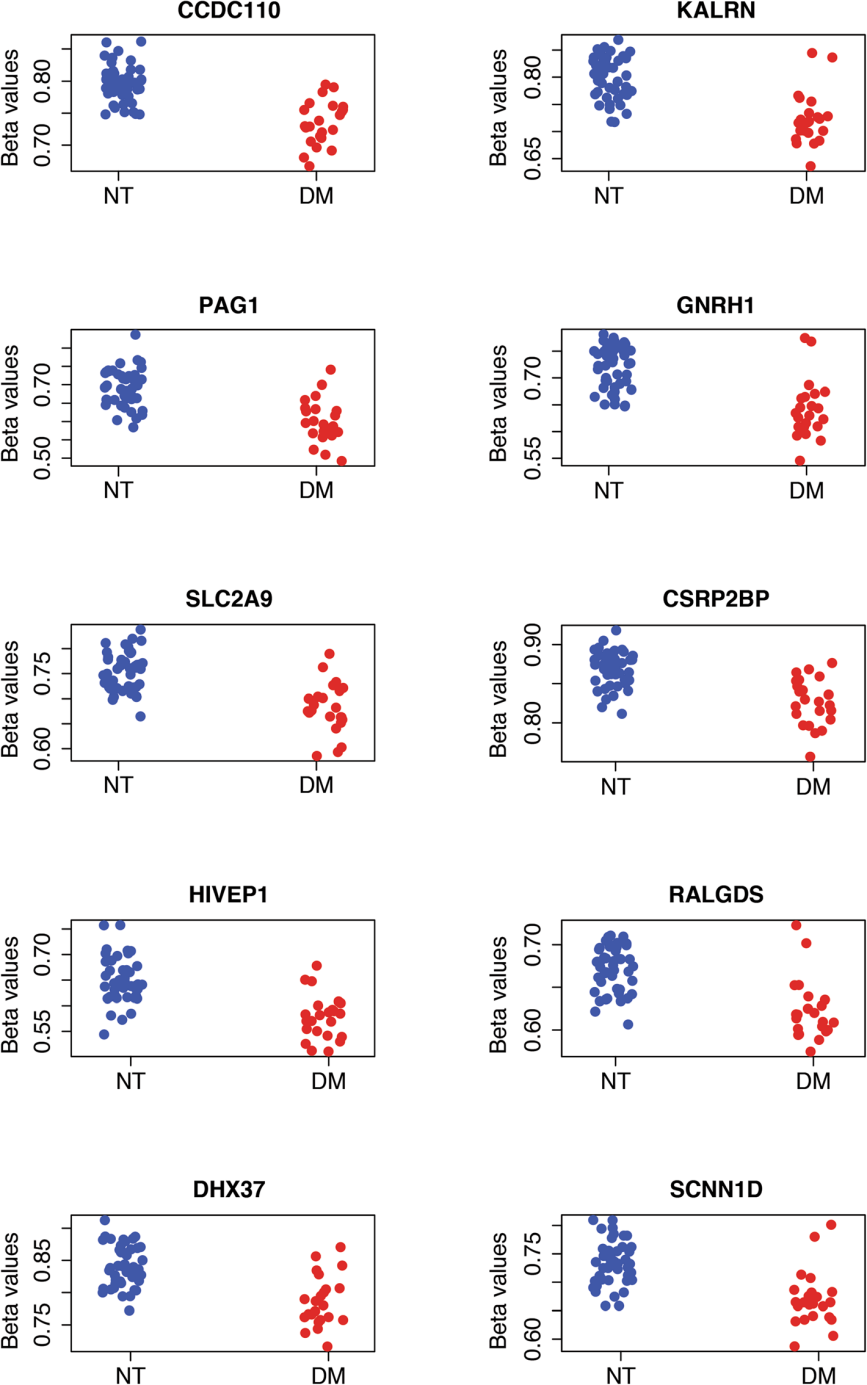
Scatter plots showing the beta value of the top 10 significantly methylated probes found by comparison between diabetic and non-diabetic groups using linear regression and empirical Bayes statistics for differential expression

Unadjusted as well as following correction for maternal age, pre-pregnancy BMI, and method of delivery, a total of 15 CpG sites showed significant differential methylation in the diabetic subgroup (Fig. 1) with the top 10 CpG mapped genes of CCDC110, KALRN, PAG1, GNRH1, SLC2A9, CSRP2BP, HIVEP1, RALGDS, DHX37, and SCNN1D highlighted in Table 2. An additional six significant (adj. *p* value < 0.05) CpG sites were identified, but lacked an associated gene (cg08242354, cg23184039, cg00866179, cg17162208, cg24798727).

**Table 2:** Top differentially methylated probes from a comparison of diabetic vs non-diabetic samples

Gene ID	cg ID	Gene name	Function/pathway	Associated disorders/diseases	Gene location	Mean difference (*M* value)	Adjusted *p* value
CCDC110	cg07221855	Coiled-coil domain-containing protein 110			Body	− 0.484995	0.000916
KALRN	cg20807374	Kalirin	Signaling receptor binding; promotes the exchange of GDP by GTP; activates specific Rho GTPase family members; regulates neuronal shape, growth, and plasticity; interacts with the huntingtin-associated protein 1	Huntington’s disease, coronary heart disease	Body	− 0.608775	0.002236
PAG1	cg01108434	Phosphoprotein associated with glycosphingolipid-enriched microdomains	Type III transmembrane adaptor protein; binds to tyrosine kinase csk protein; regulation of T cell activation		5′UTR	− 0.584836	0.002785
GNRH1	cg25710809	Progonadoliberin-1	Hormone receptor binding/activity; signaling receptor activity; gonadotropin-releasing hormone receptor pathway	Hypo-gonadotropic hypogonadism	TSS1500	− 0.511411	0.002785
SLC2A9	cg26210521	Solute carrier family 2, facilitated glucose transporter member 9	Glucose transporter 9 (GLUT9); located in proximal tubules, reabsorption of nutrients, water, and other into the blood and excretion into the urine; reabsorbing and excreting glucose	Renal hypouricemia, gout	Body	− 0.519168	0.004015
CSRP2BP	cg19354792	Cysteine-rich protein 2-binding protein	Histone acetyltransferase activity; zinc finger protein adapters, acetyltransferase domain	Cytochrome c oxidase deficiency	Body; TSS1500	− 0.463411	0.004738
HIVEP1	cg14398337	Human immunodeficiency virus type I enhancer binding protein 1/zinc finger protein 40	Transcription factor belonging, enhancer elements of several viral promoters, binds to a sequence motif, transcriptional regulation of both viral and cellular genes	OCD, ADHD	Body	− 0.427399	0.006924
RALGDS	cg10509965	RAL guanine nucleotide dissociation stimulator	Guanyl-nucleotide exchange factor, exchange of GDP and GTP in a G-protein; enzyme regulator activity; G-protein coupled receptor signaling pathway; MAPK cascade; cell cycle		Body	− 0.300234	0.018452
DHX37	cg04226314	ATP-dependent RNA helicase DHX37 related	RNA helicase; ATP-dependent helicase activity; catalytic activity, acting on RNA; rRNA processing, gene expression; DEAD box, embryogenesis, spermatogenesis, and cellular growth and division		Body	− 0.504887	0.030713
SCNN1D	cg12120973	Amiloride-sensitive sodium channel subunit delta	Ion channel; cation transport; regulation of biological processes; sensory perception of pain and taste; transport of glucose and other sugars, bile salts and organic acids, metal ions, and amine compounds		5′UTR; 1stExon; Body	− 0.430032	0.034144

A similar EWAS on obesity revealed no further epigenome-wide associated CpG sites (data not shown) using a linear modeling approach as well as including covariates (data not shown).

### Regional analysis using bumphunter

In regional analyses, we identified three regions that met *p* value < 0.05 for diabetes and three regions for obesity (Table 3). A region of four CpG sites (cg06417478, cg04657146, cg11738485, and cg23899408) is associated with HOOK2, or Hook Microtubule Tethering Protein 2, and was significantly hypomethylated compared with NT in the diabetic subgroup. Additionally, LCE3C (cg09972436; Late Cornified Envelope 3C) and TMEM63B (cg25069157; Transmembrane Protein 63B) were also hypomethylated compared with NT. LTF (cg21787089, cg01427108; Lactotransferrin) and DUSP22 (cg01516881, cg26668828 (body); cg18110333, cg05064044 (1stExon; 5′UTR); Dual Specificity Phosphatase 22) were also differentially methylated with LTF hypermethylated and DUSP22 hypomethylated respectively compared with NT in the obese subgroup.

**Table 3:** Probes and genes associated with significantly differentially methylated regions found using bumphunter analysis for both obese and diabetic comparisons

Gene ID	cg ID	Gene name	Function/pathway	Associated disorders/diseases	Gene location	Mean difference (*M* value)	*P* value	Comparison
HOOK2	cg06417478, cg04657146, cg11738485, cg23899408	Hook Microtubule Tethering Protein 2	Positioning or formation of aggresomes		Body	− 0.7415667	0.001	Diabetic
LCE3C	cg09972436	Late Cornified Envelope 3C	Keratinization and developmental biology	Psoriatic arthritis	TSS1500	− 0.8573606	0.04	Diabetic
TMEM63B	cg25069157	Transmembrane Protein 63B	Osmosensitive calcium-permeable cation channel		Body	− 0.8412484	0.04	Diabetic
LTF	cg21787089, cg01427108	Lactotransferrin	Regulation of iron homeostasis, host defense against a broad range of microbial infections, anti-inflammatory activity, regulation of cellular growth and differentiation and protection against cancer development and metastasis	Mastitis, kerato-conjunctivitis sicca, rheumatoid vasculitis, *Clostridium difficile* colitis, dental caries	Body	0.6222828	0.016	Obese
DUSP22	cg01516881, cg26668828	Dual Specificity Phosphatase 22	Activates the Jnk signaling pathway; dephosphorylates and deactivates p38 and stress-activated protein kinase/c-Jun N-terminal kinase	Alk-negative anaplastic large cell lymphoma	Body	− 0.5737223	0.029	Obese
DUSP22	cg18110333, cg05064044	Dual Specificity Phosphatase 22	Activates the Jnk signaling pathway; dephosphorylates and deactivates p38 and stress-activated protein kinase/c-Jun N-terminal kinase	Alk-negative anaplastic large cell lymphoma	1stExon; 5′UTR	− 0.5694813	0.03	Obese

### Correlation analysis with infant outcome

CpG sites identified during EWAS were directly compared with infantile demographic and body composition markers for potential phenotypic association (Table 4). No genes or cg IDs correlated with infantile birth weight. cg23184039 was associated with changes in birth length, frontal-occipital circumference, and gestational age; however, this CpG site is not associated with a gene identified. SLC2A9 and CSRP2BP correlated with gestational age.

**Table 4:** Correlation analysis with EWAS identified probes and infantile demographic

Gene ID	cg ID	Birth weight	Birth length	Gestational age	Frontal-occipital circumference
CCDC110	cg07221855	0.0316	0.1342	0.216	0.2299
KALRN	cg20807374	0.0379	0.1486	0.1914	0.1076
PAG1	cg01108434	− 0.144	0.065	0.1099	− 0.1295
GNRH1	cg25710809	− 0.0047	0.1698	0.2007	0.2128
SLC2A9	cg26210521	0.0372	0.2344	**0.2397**	0.0826
CSRP2BP	cg19354792	− 0.0779	0.1156	**0.2569**	0.0694
HIVEP1	cg14398337	− 0.0216	0.0774	0.1003	0.0971
RALGDS	cg10509965	0.0511	0.2327	0.1567	0.1666
DHX37	cg04226314	0.0675	0.1983	0.1781	0.1802
SCNN1D	cg12120973	0.1393	0.2301	0.1719	0.1544
None	cg23184039	0.1846	**0.2463**	**0.2682**	**0.2642**
None	cg24798727	− 0.036	0.1752	0.1676	0.1455
None	cg17162208	0.0308	0.1806	0.1518	0.2326
None	cg00866179	− 0.0146	0.1271	0.1459	0.0644
None	cg08242354	0.034	0.1235	0.149	0.1847

Bold are considered significant, *p* < 0.05

## Discussion

In this study, we sought to characterize how the fetal epigenome could be altered by the maternal environment, potentially predisposing the infant to long-term comorbidities of metabolic syndrome.

We identified multiple genes of interest through EWAS or regional analysis with significant differential methylation potentially caused by the presence of maternal diabetes or obesity in a largely Hispanic population. The genes identified from our population have not been previously reported in existing literature as differentially methylated in regard to maternal comorbidities during pregnancy. Genes identified are often hypomethylated when compared with the non-diabetic groups indicating potential elevated expression patterns in the newborns born to diabetic mothers, although we did not confirm gene expression profiles.

### Epigenome-wide association study

Epigenome-wide association study found 15 (*p* adj. < 0.05) significant probes in a comparison between the diabetic and non-diabetic samples (Additional file 1); all 15 probes were hypomethylated compared with the non-diabetic group. No significant probes were found in a comparison between the NT and OB groups. Of the probes found in the DM comparison, ten are known to be associated with genes; the other five have no currently known gene association. A review of the genes associated with the significant probes found that several of the genes were associated with diabetes and/or obesity.

The SLC2A9 gene produces GLUT-9, a transport protein which facilitates the transport of glucose, fructose, and other sugars, and expression is specifically localized to insulin-containing β cells regulating glucose-stimulated insulin secretion [32]. In a study that looked at the levels of glucose transporter expression in placental tissue from mothers with diabetes mellitus, they found there was a significant increase in the expression of GLUT-9 in diabetic mothers controlled by insulin, as well as pregestational diabetes [33].

KALRN, or Kalirin RhoGEF Kinase, is a guanine exchange factor which acts on several Rho GTPases. Potential diseases associated with this gene include Huntington’s disease, coronary heart disease, various cardiovascular disorders, and ischemic stroke possibly related to nitric oxide signaling pathways [34].

GNRH1 encodes the precursor to gonadotropin-releasing hormone-1 (GnRH1). The effect of DM on GnRH expression is associated with the regulation of B and T cell response in pregnancy. One study found that antibodies against GnRH1, LH, and other related hormones were present more often in patients with diabetes mellitus [35].

RALGDS encodes a guanylyl-nucleotide exchange factor (GEFs) specifically involved in signal transduction pathways regulating cell growth and cancer/tumorigenesis in humans [36]. In addition, RalGDS activates Akt kinase whose abnormal expression is implicated in diabetes mellitus pathology [37, 38]. Akt interacts with insulin receptor substrate 1, PI3K, and GLUT4 translocation during insulin stimulation, as well as inactivates glycogen-synthase kinase-3, promoting glycogen synthesis [38].

### Regional association study (DM)

Regional association study between DM and non-DM found three regions that were significant between the two comparisons. Of the most relevant to this study, four probes in a region associated with the gene HOOK2 were found to be hypomethylated in the DM group compared with the non-DM group. HOOK2 belongs to the HOOK family of proteins, which are responsible for trafficking and anchoring of organelles in the cell through the binding and directing of microtubules [36]. A study of DNA methylation in adipose tissue from subjects with type II diabetes and obesity, using the 450K Illumina beadchip, identified HOOK2 as significantly differentially methylated from the healthy group, although their findings indicated that HOOK2 was hypermethylated while our results show the genes being hypomethylated [39]. In an additional DNA methylation study on mothers with gestational diabetes, HOOK2 was found to be commonly differentially methylated in the maternal blood, placenta, and umbilical cord [40].

### Regional association study (OB)

Three regions were identified as significantly differentially methylated between OB and non-OB groups using a regional association study. Two of the probes identified are associated with the gene LTF, which encodes lactotransferrin (Lf). Lf is a member of the iron-binding protein transferrin family and is involved in the regulation of iron homeostasis, anti-inflammatory response, cell growth regulation, differentiation, innate immune response, and antimicrobial activity [36]. Lf has also been shown to be positively correlated in individuals diagnosed with insulin resistance and type II diabetes and negatively correlated with body adiposity [41]. Moreno-Navarrete et al. confirmed these results, finding a decrease of Lf in hyperglycemic and obese individuals and an increase in insulin sensitive adults [42].

Four of the probes identified are associated with the gene DUSP22, encoding dual specificity phosphatase 22, or JNK pathway-associated phosphatase, and implicated in insulin receptor phosphorylation [36]. DUSP22 also represses the activation of T cells by phosphorylation of Lck, a Src-family tyrosine kinase involved in activation of T cell receptors during adaptive immune response. DUSP22 knockout mice were found to have depressed immune response, and later in life increased autoantibodies [43].

### Correlation analysis with infant outcome

There were no identified CpG sites associated with birth weight changes. SLC2A9, or the GLUT-9 transport protein and CSRP2BP, a Cysteine-rich protein 2-binding protein correlated with gestational age. It is unclear the phenotypic significance of these associations as no changes were noted in the infant body composition anthropometrics. Interestingly, cg23184039, which has not been associated with a gene ID, was associated with increased birth length, frontal-occipital circumference, and gestational age in our population. This could potentially represent a future target for larger studies including expression and body composition beyond infancy.

### Limitations

It is important to note that we have not shown experimentally that the methylation status of these significant genes has a phenotypic effect, only that there is a correlation between the conditions of obesity and diabetes and the differential methylation identified in the 69 samples. While we acknowledge the small sample size evaluated, the population of this study was almost exclusively comprised of mothers of Hispanic ethnicity, as such it should be noted that our results may reflect a more narrowed analysis of the epigenetic effects of maternal diabetes and obesity which is specific to populations of Hispanic ethnicity. Additionally, the identified genes detected through probe wise and peak detection and their differential methylation may or may not be associated with altered protein expression, which was not determined during this project. Without evaluation of protein expression or additional biomarkers, we are unable to potentially link these effects on phenotype or potential development of obesity or diabetes in the offspring beyond neonatal body composition.

## Conclusion

Differential DNA methylation in the fetal epigenome is associated with exposure to maternal obesity and diabetes mellitus in a highly Hispanic population. DNA methylation of genes identified such as SLC2A9, HOOK2, LTF, and DUSP22 all have direct or indirect links to diabetes or obesity including immune or inflammatory regulatory pathways, signaling pathways, and clinical disorders related to diabetes and obesity. Future prospective studies are needed to assess the effects of maternal obesity and diabetes and its differential methylation effects on protein expression and offspring phenotypic effect including body composition and clinical risk of acquired disease in this high-risk population.

## Supplementary information

**Additional file 1 Fig. S1.** Manhattan Plots of CpG sites for differentially methylated sites from either non-diabetic vs. diabetic (Panel A) or non-obese vs obese (Panel B) models. Unadjusted *p*-values models without correction for covariates is presented.

## Data Availability

The datasets generated and/or analyzed during the current study are not publicly available due to legal restrictions but are available from the corresponding author on reasonable request.

## Abbreviations

DM: Diabetes mellitus
DNA: Deoxyribonucleic acid
EWAS: Epigenome-wide association study
OB: Obesity
QC: Quality control
SD: Standard deviation
SNP: Single-nucleotide polymorphism
