# Supplementary material for: Maternal diabetes and obesity influence the fetal epigenome in a largely Hispanic population
Source: Clin Epigenetics. 2020 Feb 19;12:34. doi: 10.1186/s13148-020-0824-9 (PMC7031937; doi:10.1186/s13148-020-0824-9)
Supplement: Supplementary file 1 — Additional file 1 Fig. S1. Manhattan Plots of CpG sites for differentially methylated sites from either non-diabetic vs. diabetic (Panel A) or non-obese vs obese (Panel B) models. Unadjusted p-values models without correction for covariates is presented. [file 13148_2020_824_MOESM1_ESM.docx]

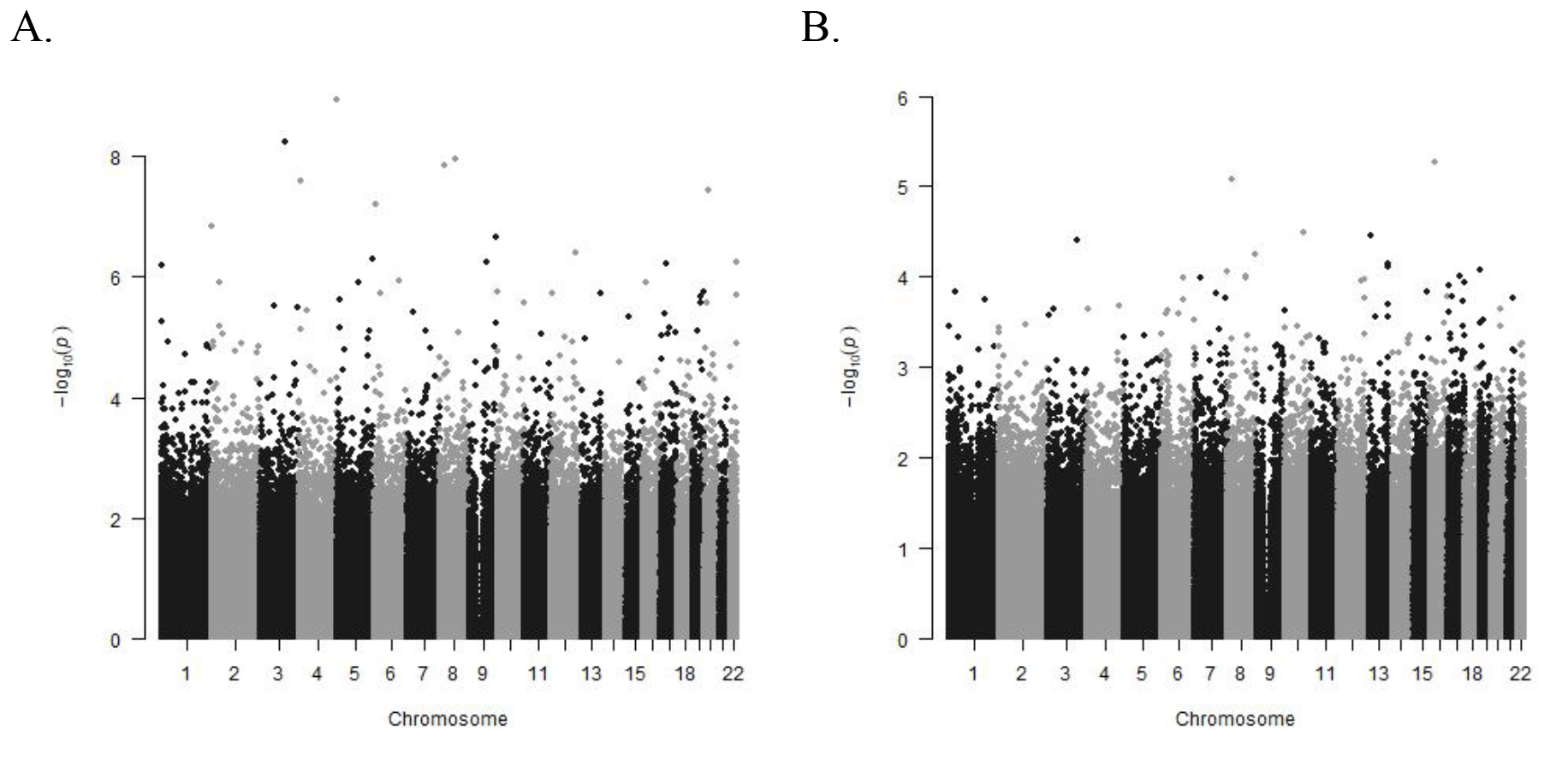


Supplementary Figure 1:

Manhattan Plots of CpG sites for differentially methylated sites from either non-diabetic vs. diabetic (Panel A) or non-obese vs obese (Panel B) models. Unadjusted p-values models without correction for covariates is presented.
